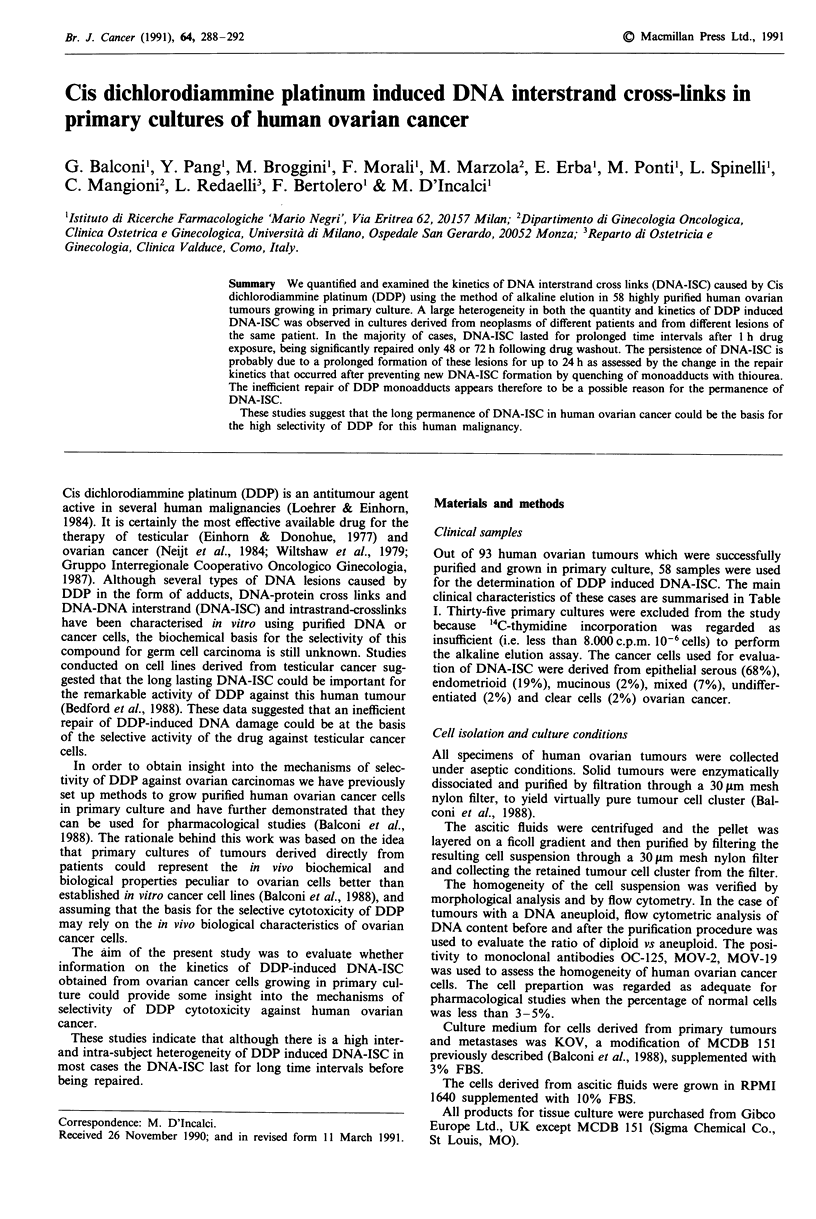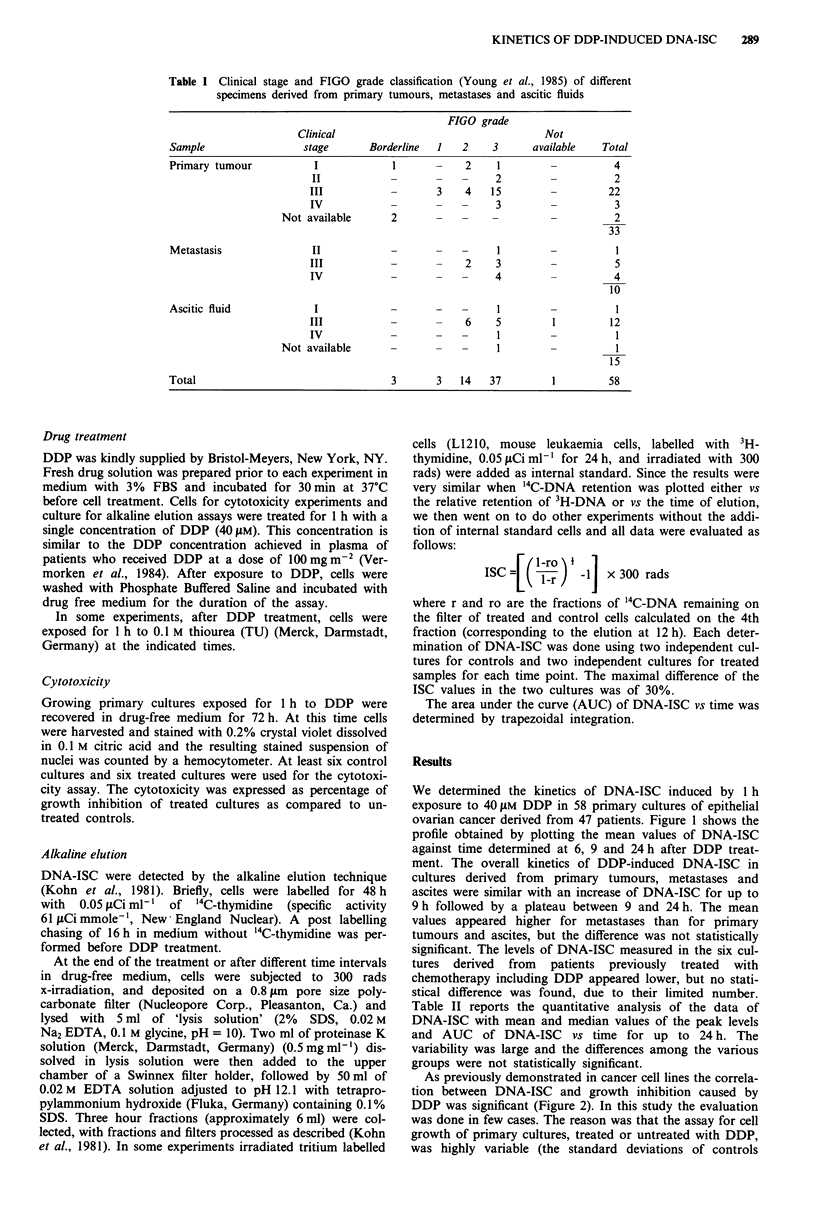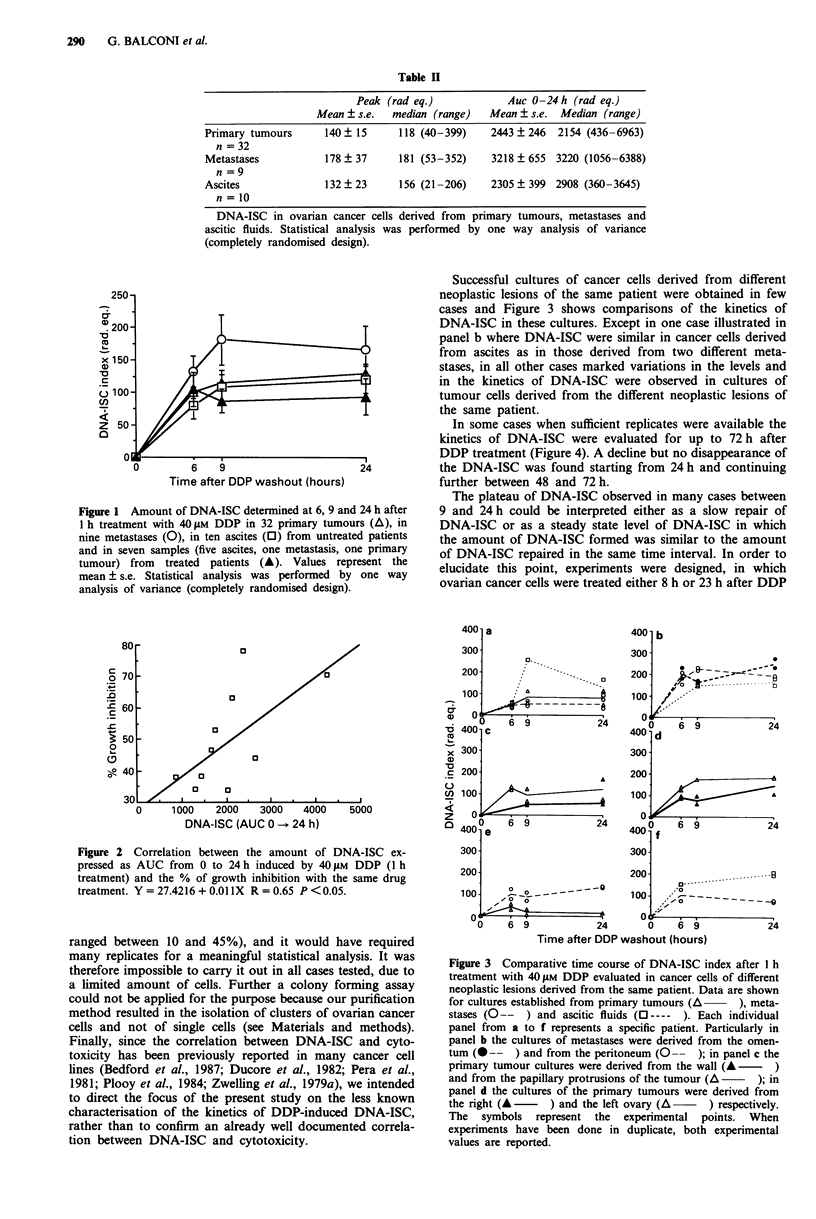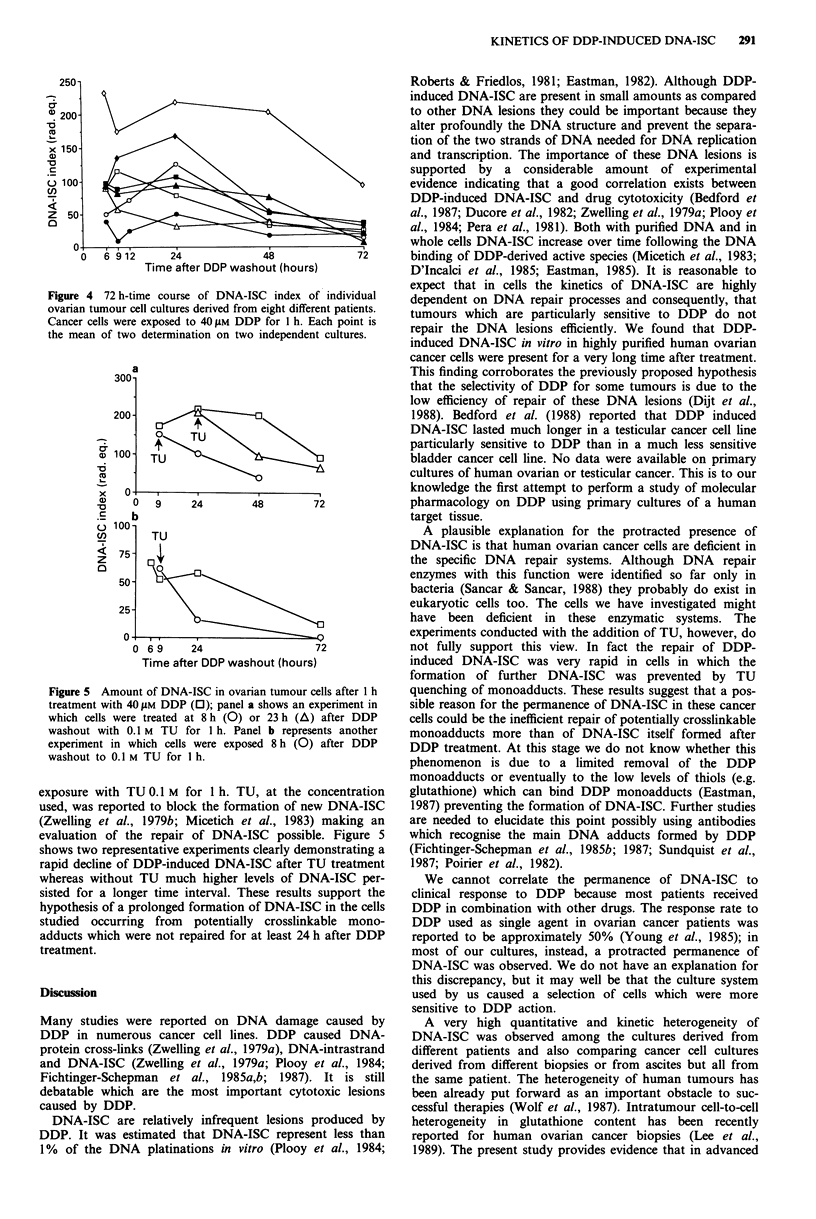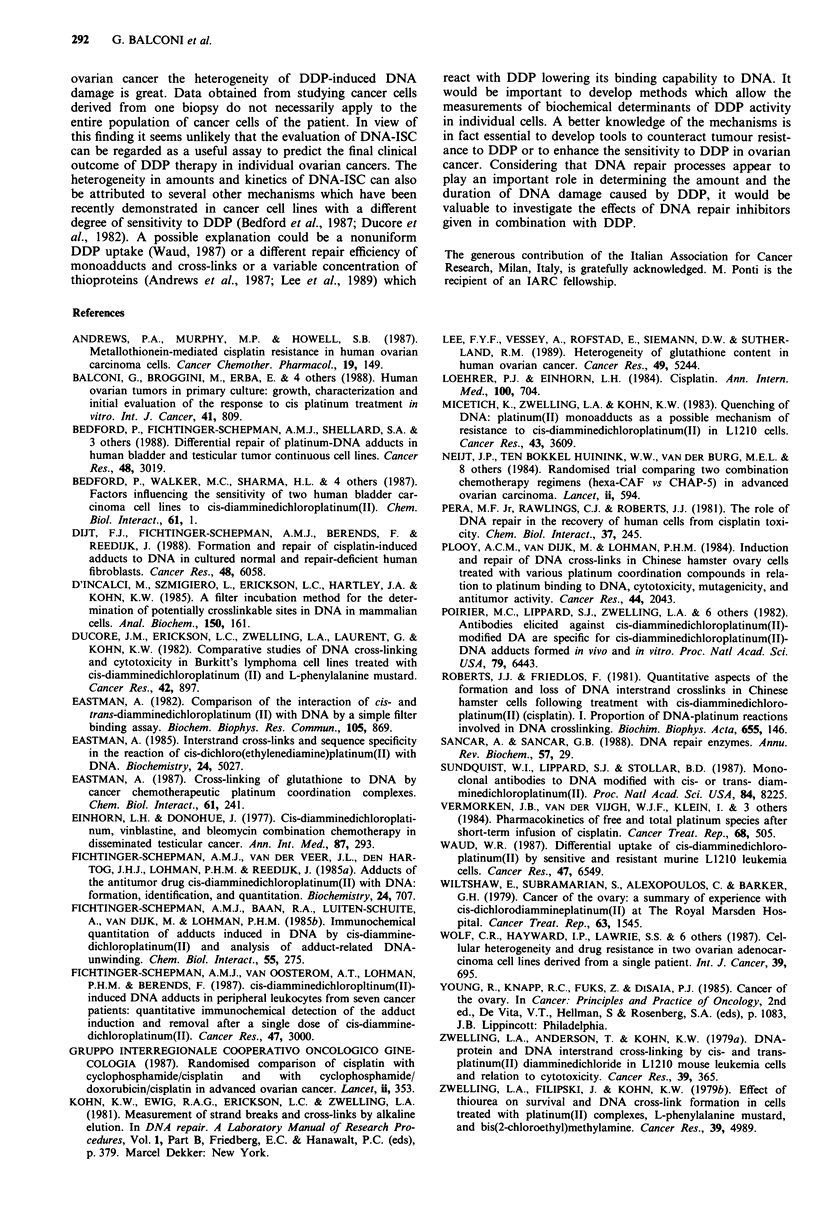# Cis dichlorodiammine platinum induced DNA interstrand cross-links in primary cultures of human ovarian cancer.

**DOI:** 10.1038/bjc.1991.293

**Published:** 1991-08

**Authors:** G. Balconi, Y. Pang, M. Broggini, F. Morali, M. Marzola, E. Erba, M. Ponti, L. Spinelli, C. Mangioni, L. Redaelli

**Affiliations:** Istituto di Ricerche Farmacologiche Mario Negri, Milan, Italy.

## Abstract

We quantified and examined the kinetics of DNA interstrand cross links (DNA-ISC) caused by Cis dichlorodiammine platinum (DDP) using the method of alkaline elution in 58 highly purified human ovarian tumours growing in primary culture. A large heterogeneity in both the quantity and kinetics of DDP induced DNA-ISC was observed in cultures derived from neoplasms of different patients and from different lesions of the same patient. In the majority of cases. DNA-ISC lasted for prolonged time intervals after 1 h drug exposure, being significantly repaired only 48 or 72 h following drug washout. The persistence of DNA-ISC is probably due to a prolonged formation of these lesions for up to 24 h as assessed by the change in the repair kinetics that occurred after preventing new DNA-ISC formation by quenching of monoadducts with thiourea. The inefficient repair of DDP monoadducts appears therefore to be a possible reason for the permanence of DNA-ISC. These studies suggest that the long permanence of DNA-ISC in human ovarian cancer could be the basis for the high selectivity of DDP for this human malignancy.


© Macmillan Press Ltd., 1991

### Cis dichlorodiammine platinum induced DNA interstrand cross-links in primary cultures of human ovarian cancer

G. Balconi[1], Y. Pang[1], M. Broggini[1], F. Morali[1], M. Marzola[2], E. Erba[1], M. Ponti[1], L. Spinelli[1], C. Mangioni[2], L. Redaelli[3], F. Bertolero[1] & M. D'Incalci[1]

[1]*Istituto di Ricerche Farmacologiche 'Mario Negri', Via Eritrea 62, 20157 Milan;* [2]*Dipartimento di Ginecologia Oncologica, Clinica Ostetrica e Ginecologica, Università di Milano, Ospedale San Gerardo, 20052 Monza;* [3]*Reparto di Ostetricia e Ginecologia, Clinica Valduce, Como, Italy.*

**Summary** We quantified and examined the kinetics of DNA interstrand cross links (DNA-ISC) caused by Cis dichlorodiammine platinum (DDP) using the method of alkaline elution in 58 highly purified human ovarian tumours growing in primary culture. A large heterogeneity in both the quantity and kinetics of DDP induced DNA-ISC was observed in cultures derived from neoplasms of different patients and from different lesions of the same patient. In the majority of cases, DNA-ISC lasted for prolonged time intervals after 1 h drug exposure, being significantly repaired only 48 or 72 h following drug washout. The persistence of DNA-ISC is probably due to a prolonged formation of these lesions for up to 24 h as assessed by the change in the repair kinetics that occurred after preventing new DNA-ISC formation by quenching of monoadducts with thiourea. The inefficient repair of DDP monoadducts appears therefore to be a possible reason for the permanence of DNA-ISC.

These studies suggest that the long permanence of DNA-ISC in human ovarian cancer could be the basis for the high selectivity of DDP for this human malignancy.

Cis dichlorodiammine platinum (DDP) is an antitumour agent active in several human malignancies (Loehrer & Einhorn, 1984). It is certainly the most effective available drug for the therapy of testicular (Einhorn & Donohue, 1977) and ovarian cancer (Neijt *et al.*, 1984; Wiltshaw *et al.*, 1979; Gruppo Interregionale Cooperativo Oncologico Ginecologia, 1987). Although several types of DNA lesions caused by DDP in the form of adducts, DNA-protein cross links and DNA-DNA interstrand (DNA-ISC) and intrastrand-crosslinks have been characterised *in vitro* using purified DNA or cancer cells, the biochemical basis for the selectivity of this compound for germ cell carcinoma is still unknown. Studies conducted on cell lines derived from testicular cancer suggested that the long lasting DNA-ISC could be important for the remarkable activity of DDP against this human tumour (Bedford *et al.*, 1988). These data suggested that an inefficient repair of DDP-induced DNA damage could be at the basis of the selective activity of the drug against testicular cancer cells.

In order to obtain insight into the mechanisms of selectivity of DDP against ovarian carcinomas we have previously set up methods to grow purified human ovarian cancer cells in primary culture and have further demonstrated that they can be used for pharmacological studies (Balconi *et al.*, 1988). The rationale behind this work was based on the idea that primary cultures of tumours derived directly from patients could represent the *in vivo* biochemical and biological properties peculiar to ovarian cells better than established *in vitro* cancer cell lines (Balconi *et al.*, 1988), and assuming that the basis for the selective cytotoxicity of DDP may rely on the *in vivo* biological characteristics of ovarian cancer cells.

The aim of the present study was to evaluate whether information on the kinetics of DDP-induced DNA-ISC obtained from ovarian cancer cells growing in primary culture could provide some insight into the mechanisms of selectivity of DDP cytotoxicity against human ovarian cancer.

These studies indicate that although there is a high inter- and intra-subject heterogeneity of DDP induced DNA-ISC in most cases the DNA-ISC last for long time intervals before being repaired.

Correspondence: M. D'Incalci.
Received 26 November 1990; and in revised form 11 March 1991.

### Materials and methods

### *Clinical samples*

Out of 93 human ovarian tumours which were successfully purified and grown in primary culture, 58 samples were used for the determination of DDP induced DNA-ISC. The main clinical characteristics of these cases are summarised in Table I. Thirty-five primary cultures were excluded from the study because $^{14}C$-thymidine incorporation was regarded as insufficient (i.e. less than 8.000 c.p.m. $10^{-6}$ cells) to perform the alkaline elution assay. The cancer cells used for evaluation of DNA-ISC were derived from epithelial serous (68%), endometrioid (19%), mucinous (2%), mixed (7%), undifferentiated (2%) and clear cells (2%) ovarian cancer.

### *Cell isolation and culture conditions*

All specimens of human ovarian tumours were collected under aseptic conditions. Solid tumours were enzymatically dissociated and purified by filtration through a 30 $\mu m$ mesh nylon filter, to yield virtually pure tumour cell cluster (Balconi *et al.*, 1988).

The ascitic fluids were centrifuged and the pellet was layered on a ficoll gradient and then purified by filtering the resulting cell suspension through a 30 $\mu m$ mesh nylon filter and collecting the retained tumour cell cluster from the filter.

The homogeneity of the cell suspension was verified by morphological analysis and by flow cytometry. In the case of tumours with a DNA aneuploid, flow cytometric analysis of DNA content before and after the purification procedure was used to evaluate the ratio of diploid *vs* aneuploid. The positivity to monoclonal antibodies OC-125, MOV-2, MOV-19 was used to assess the homogeneity of human ovarian cancer cells. The cell prepartion was regarded as adequate for pharmacological studies when the percentage of normal cells was less than 3-5%.

Culture medium for cells derived from primary tumours and metastases was KOV, a modification of MCDB 151 previously described (Balconi *et al.*, 1988), supplemented with 3% FBS.

The cells derived from ascitic fluids were grown in RPMI 1640 supplemented with 10% FBS.

All products for tissue culture were purchased from Gibco Europe Ltd., UK except MCDB 151 (Sigma Chemical Co., St Louis, MO).

**Table I** Clinical stage and FIGO grade classification (Young *et al.*, 1985) of different specimens derived from primary tumours, metastases and ascitic fluids

| Sample | Clinical stage | FIGO grade: Borderline | 1 | 2 | 3 | Not available | Total |
|---|---|---|---|---|---|---|---|
| Primary tumour | I | 1 | – | 2 | 1 | – | 4 |
| | II | – | – | – | 2 | – | 2 |
| | III | – | 3 | 4 | 15 | – | 22 |
| | IV | – | – | – | 3 | – | 3 |
| | Not available | 2 | – | – | – | – | 2 |
| | | | | | | | 33 |
| Metastasis | II | – | – | – | 1 | – | 1 |
| | III | – | – | 2 | 3 | – | 5 |
| | IV | – | – | – | 4 | – | 4 |
| | | | | | | | 10 |
| Ascitic fluid | I | – | – | – | 1 | – | 1 |
| | III | – | – | 6 | 5 | 1 | 12 |
| | IV | – | – | – | 1 | – | 1 |
| | Not available | – | – | – | 1 | – | 1 |
| | | | | | | | 15 |
| Total | | 3 | 3 | 14 | 37 | 1 | 58 |

*Drug treatment*

DDP was kindly supplied by Bristol-Meyers, New York, NY. Fresh drug solution was prepared prior to each experiment in medium with 3% FBS and incubated for 30 min at 37°C before cell treatment. Cells for cytotoxicity experiments and culture for alkaline elution assays were treated for 1 h with a single concentration of DDP (40 μM). This concentration is similar to the DDP concentration achieved in plasma of patients who received DDP at a dose of 100 mg $m^{-2}$ (Vermorken *et al.*, 1984). After exposure to DDP, cells were washed with Phosphate Buffered Saline and incubated with drug free medium for the duration of the assay.

In some experiments, after DDP treatment, cells were exposed for 1 h to 0.1 M thiourea (TU) (Merck, Darmstadt, Germany) at the indicated times.

*Cytotoxicity*

Growing primary cultures exposed for 1 h to DDP were recovered in drug-free medium for 72 h. At this time cells were harvested and stained with 0.2% crystal violet dissolved in 0.1 M citric acid and the resulting stained suspension of nuclei was counted by a hemocytometer. At least six control cultures and six treated cultures were used for the cytotoxicity assay. The cytotoxicity was expressed as percentage of growth inhibition of treated cultures as compared to untreated controls.

*Alkaline elution*

DNA-ISC were detected by the alkaline elution technique (Kohn *et al.*, 1981). Briefly, cells were labelled for 48 h with 0.05 μCi $ml^{-1}$ of $^{14}$C-thymidine (specific activity 61 μCi $mmole^{-1}$, New England Nuclear). A post labelling chasing of 16 h in medium without $^{14}$C-thymidine was performed before DDP treatment.

At the end of the treatment or after different time intervals in drug-free medium, cells were subjected to 300 rads x-irradiation, and deposited on a 0.8 μm pore size polycarbonate filter (Nucleopore Corp., Pleasanton, Ca.) and lysed with 5 ml of 'lysis solution' (2% SDS, 0.02 M $Na_2$ EDTA, 0.1 M glycine, pH = 10). Two ml of proteinase K solution (Merck, Darmstadt, Germany) (0.5 mg $ml^{-1}$) dissolved in lysis solution were then added to the upper chamber of a Swinnex filter holder, followed by 50 ml of 0.02 M EDTA solution adjusted to pH 12.1 with tetrapropylammonium hydroxide (Fluka, Germany) containing 0.1% SDS. Three hour fractions (approximately 6 ml) were collected, with fractions and filters processed as described (Kohn *et al.*, 1981). In some experiments irradiated tritium labelled cells (L1210, mouse leukaemia cells, labelled with $^3$H-thymidine, 0.05 μCi $ml^{-1}$ for 24 h, and irradiated with 300 rads) were added as internal standard. Since the results were very similar when $^{14}$C-DNA retention was plotted either *vs* the relative retention of $^3$H-DNA or *vs* the time of elution, we then went on to do other experiments without the addition of internal standard cells and all data were evaluated as follows:

$$ISC = \left[\left(\frac{1\text{-ro}}{1\text{-r}}\right)^{\frac{1}{2}} - 1\right] \times 300 \text{ rads}$$

where r and ro are the fractions of $^{14}$C-DNA remaining on the filter of treated and control cells calculated on the 4th fraction (corresponding to the elution at 12 h). Each determination of DNA-ISC was done using two independent cultures for controls and two independent cultures for treated samples for each time point. The maximal difference of the ISC values in the two cultures was of 30%.

The area under the curve (AUC) of DNA-ISC *vs* time was determined by trapezoidal integration.

### Results

We determined the kinetics of DNA-ISC induced by 1 h exposure to 40 μM DDP in 58 primary cultures of epithelial ovarian cancer derived from 47 patients. Figure 1 shows the profile obtained by plotting the mean values of DNA-ISC against time determined at 6, 9 and 24 h after DDP treatment. The overall kinetics of DDP-induced DNA-ISC in cultures derived from primary tumours, metastases and ascites were similar with an increase of DNA-ISC for up to 9 h followed by a plateau between 9 and 24 h. The mean values appeared higher for metastases than for primary tumours and ascites, but the difference was not statistically significant. The levels of DNA-ISC measured in the six cultures derived from patients previously treated with chemotherapy including DDP appeared lower, but no statistical difference was found, due to their limited number. Table II reports the quantitative analysis of the data of DNA-ISC with mean and median values of the peak levels and AUC of DNA-ISC *vs* time for up to 24 h. The variability was large and the differences among the various groups were not statistically significant.

As previously demonstrated in cancer cell lines the correlation between DNA-ISC and growth inhibition caused by DDP was significant (Figure 2). In this study the evaluation was done in few cases. The reason was that the assay for cell growth of primary cultures, treated or untreated with DDP, was highly variable (the standard deviations of controls

**Table II**

| | Peak (rad eq.) Mean ± s.e. | median (range) | Auc 0–24 h (rad eq.) Mean ± s.e. | Median (range) |
|---|---|---|---|---|
| Primary tumours $n = 32$ | 140 ± 15 | 118 (40–399) | 2443 ± 246 | 2154 (436–6963) |
| Metastases $n = 9$ | 178 ± 37 | 181 (53–352) | 3218 ± 655 | 3220 (1056–6388) |
| Ascites $n = 10$ | 132 ± 23 | 156 (21–206) | 2305 ± 399 | 2908 (360–3645) |

DNA-ISC in ovarian cancer cells derived from primary tumours, metastases and ascitic fluids. Statistical analysis was performed by one way analysis of variance (completely randomised design).

**Figure 1** Amount of DNA-ISC determined at 6, 9 and 24 h after 1 h treatment with 40 μM DDP in 32 primary tumours (△), in nine metastases (○), in ten ascites (□) from untreated patients and in seven samples (five ascites, one metastasis, one primary tumour) from treated patients (▲). Values represent the mean ± s.e. Statistical analysis was performed by one way analysis of variance (completely randomised design).

**Figure 2** Correlation between the amount of DNA-ISC expressed as AUC from 0 to 24 h induced by 40 μM DDP (1 h treatment) and the % of growth inhibition with the same drug treatment. Y = 27.4216 + 0.011X R = 0.65 $P < 0.05$.

ranged between 10 and 45%), and it would have required many replicates for a meaningful statistical analysis. It was therefore impossible to carry it out in all cases tested, due to a limited amount of cells. Further a colony forming assay could not be applied for the purpose because our purification method resulted in the isolation of clusters of ovarian cancer cells and not of single cells (see Materials and methods). Finally, since the correlation between DNA-ISC and cytotoxicity has been previously reported in many cancer cell lines (Bedford *et al.*, 1987; Ducore *et al.*, 1982; Pera *et al.*, 1981; Plooy *et al.*, 1984; Zwelling *et al.*, 1979*a*), we intended to direct the focus of the present study on the less known characterisation of the kinetics of DDP-induced DNA-ISC, rather than to confirm an already well documented correlation between DNA-ISC and cytotoxicity.

Successful cultures of cancer cells derived from different neoplastic lesions of the same patient were obtained in few cases and Figure 3 shows comparisons of the kinetics of DNA-ISC in these cultures. Except in one case illustrated in panel b where DNA-ISC were similar in cancer cells derived from ascites as in those derived from two different metastases, in all other cases marked variations in the levels and in the kinetics of DNA-ISC were observed in cultures of tumour cells derived from the different neoplastic lesions of the same patient.

In some cases when sufficient replicates were available the kinetics of DNA-ISC were evaluated for up to 72 h after DDP treatment (Figure 4). A decline but no disappearance of the DNA-ISC was found starting from 24 h and continuing further between 48 and 72 h.

The plateau of DNA-ISC observed in many cases between 9 and 24 h could be interpreted either as a slow repair of DNA-ISC or as a steady state level of DNA-ISC in which the amount of DNA-ISC formed was similar to the amount of DNA-ISC repaired in the same time interval. In order to elucidate this point, experiments were designed, in which ovarian cancer cells were treated either 8 h or 23 h after DDP

**Figure 3** Comparative time course of DNA-ISC index after 1 h treatment with 40 μM DDP evaluated in cancer cells of different neoplastic lesions derived from the same patient. Data are shown for cultures established from primary tumours (△—— ), metastases (○-- ) and ascitic fluids (□---- ). Each individual panel from **a** to **f** represents a specific patient. Particularly in panel **b** the cultures of metastases were derived from the omentum (●-- ) and from the peritoneum (○-- ); in panel **c** the primary tumour cultures were derived from the wall (▲—— ) and from the papillary protrusions of the tumour (△—— ); in panel **d** the cultures of the primary tumours were derived from the right (▲—— ) and the left ovary (△—— ) respectively. The symbols represent the experimental points. When experiments have been done in duplicate, both experimental values are reported.

**Figure 4** 72 h-time course of DNA-ISC index of individual ovarian tumour cell cultures derived from eight different patients. Cancer cells were exposed to 40 µM DDP for 1 h. Each point is the mean of two determination on two independent cultures.

**Figure 5** Amount of DNA-ISC in ovarian tumour cells after 1 h treatment with 40 µM DDP (□); panel **a** shows an experiment in which cells were treated at 8 h (O) or 23 h (Δ) after DDP washout with 0.1 M TU for 1 h. Panel **b** represents another experiment in which cells were exposed 8 h (O) after DDP washout to 0.1 M TU for 1 h.

exposure with TU 0.1 M for 1 h. TU, at the concentration used, was reported to block the formation of new DNA-ISC (Zwelling *et al.*, 1979*b*; Micetich *et al.*, 1983) making an evaluation of the repair of DNA-ISC possible. Figure 5 shows two representative experiments clearly demonstrating a rapid decline of DDP-induced DNA-ISC after TU treatment whereas without TU much higher levels of DNA-ISC persisted for a longer time interval. These results support the hypothesis of a prolonged formation of DNA-ISC in the cells studied occurring from potentially crosslinkable monoadducts which were not repaired for at least 24 h after DDP treatment.

### Discussion

Many studies were reported on DNA damage caused by DDP in numerous cancer cell lines. DDP caused DNA-protein cross-links (Zwelling *et al.*, 1979*a*), DNA-intrastrand and DNA-ISC (Zwelling *et al.*, 1979*a*; Plooy *et al.*, 1984; Fichtinger-Schepman *et al.*, 1985*a*,*b*; 1987). It is still debatable which are the most important cytotoxic lesions caused by DDP.

DNA-ISC are relatively infrequent lesions produced by DDP. It was estimated that DNA-ISC represent less than 1% of the DNA platinations *in vitro* (Plooy *et al.*, 1984; Roberts & Friedlos, 1981; Eastman, 1982). Although DDP-induced DNA-ISC are present in small amounts as compared to other DNA lesions they could be important because they alter profoundly the DNA structure and prevent the separation of the two strands of DNA needed for DNA replication and transcription. The importance of these DNA lesions is supported by a considerable amount of experimental evidence indicating that a good correlation exists between DDP-induced DNA-ISC and drug cytotoxicity (Bedford *et al.*, 1987; Ducore *et al.*, 1982; Zwelling *et al.*, 1979*a*; Plooy *et al.*, 1984; Pera *et al.*, 1981). Both with purified DNA and in whole cells DNA-ISC increase over time following the DNA binding of DDP-derived active species (Micetich *et al.*, 1983; D'Incalci *et al.*, 1985; Eastman, 1985). It is reasonable to expect that in cells the kinetics of DNA-ISC are highly dependent on DNA repair processes and consequently, that tumours which are particularly sensitive to DDP do not repair the DNA lesions efficiently. We found that DDP-induced DNA-ISC *in vitro* in highly purified human ovarian cancer cells were present for a very long time after treatment. This finding corroborates the previously proposed hypothesis that the selectivity of DDP for some tumours is due to the low efficiency of repair of these DNA lesions (Dijt *et al.*, 1988). Bedford *et al.* (1988) reported that DDP induced DNA-ISC lasted much longer in a testicular cancer cell line particularly sensitive to DDP than in a much less sensitive bladder cancer cell line. No data were available on primary cultures of human ovarian or testicular cancer. This is to our knowledge the first attempt to perform a study of molecular pharmacology on DDP using primary cultures of a human target tissue.

A plausible explanation for the protracted presence of DNA-ISC is that human ovarian cancer cells are deficient in the specific DNA repair systems. Although DNA repair enzymes with this function were identified so far only in bacteria (Sancar & Sancar, 1988) they probably do exist in eukaryotic cells too. The cells we have investigated might have been deficient in these enzymatic systems. The experiments conducted with the addition of TU, however, do not fully support this view. In fact the repair of DDP-induced DNA-ISC was very rapid in cells in which the formation of further DNA-ISC was prevented by TU quenching of monoadducts. These results suggest that a possible reason for the permanence of DNA-ISC in these cancer cells could be the inefficient repair of potentially crosslinkable monoadducts more than of DNA-ISC itself formed after DDP treatment. At this stage we do not know whether this phenomenon is due to a limited removal of the DDP monoadducts or eventually to the low levels of thiols (e.g. glutathione) which can bind DDP monoadducts (Eastman, 1987) preventing the formation of DNA-ISC. Further studies are needed to elucidate this point possibly using antibodies which recognise the main DNA adducts formed by DDP (Fichtinger-Schepman *et al.*, 1985*b*; 1987; Sundquist *et al.*, 1987; Poirier *et al.*, 1982).

We cannot correlate the permanence of DNA-ISC to clinical response to DDP because most patients received DDP in combination with other drugs. The response rate to DDP used as single agent in ovarian cancer patients was reported to be approximately 50% (Young *et al.*, 1985); in most of our cultures, instead, a protracted permanence of DNA-ISC was observed. We do not have an explanation for this discrepancy, but it may well be that the culture system used by us caused a selection of cells which were more sensitive to DDP action.

A very high quantitative and kinetic heterogeneity of DNA-ISC was observed among the cultures derived from different patients and also comparing cancer cell cultures derived from different biopsies or from ascites but all from the same patient. The heterogeneity of human tumours has been already put forward as an important obstacle to successful therapies (Wolf *et al.*, 1987). Intratumour cell-to-cell heterogeneity in glutathione content has been recently reported for human ovarian cancer biopsies (Lee *et al.*, 1989). The present study provides evidence that in advanced ovarian cancer the heterogeneity of DDP-induced DNA damage is great. Data obtained from studying cancer cells derived from one biopsy do not necessarily apply to the entire population of cancer cells of the patient. In view of this finding it seems unlikely that the evaluation of DNA-ISC can be regarded as a useful assay to predict the final clinical outcome of DDP therapy in individual ovarian cancers. The heterogeneity in amounts and kinetics of DNA-ISC can also be attributed to several other mechanisms which have been recently demonstrated in cancer cell lines with a different degree of sensitivity to DDP (Bedford *et al.*, 1987; Ducore *et al.*, 1982). A possible explanation could be a nonuniform DDP uptake (Waud, 1987) or a different repair efficiency of monoadducts and cross-links or a variable concentration of thioproteins (Andrews *et al.*, 1987; Lee *et al.*, 1989) which react with DDP lowering its binding capability to DNA. It would be important to develop methods which allow the measurements of biochemical determinants of DDP activity in individual cells. A better knowledge of the mechanisms is in fact essential to develop tools to counteract tumour resistance to DDP or to enhance the sensitivity to DDP in ovarian cancer. Considering that DNA repair processes appear to play an important role in determining the amount and the duration of DNA damage caused by DDP, it would be valuable to investigate the effects of DNA repair inhibitors given in combination with DDP.

The generous contribution of the Italian Association for Cancer Research, Milan, Italy, is gratefully acknowledged. M. Ponti is the recipient of an IARC fellowship.

**References**

ANDREWS, P.A., MURPHY, M.P. & HOWELL, S.B. (1987). Metallothionein-mediated cisplatin resistance in human ovarian carcinoma cells. *Cancer Chemother. Pharmacol.*, **19**, 149.

BALCONI, G., BROGGINI, M., ERBA, E. & 4 others (1988). Human ovarian tumors in primary culture: growth, characterization and initial evaluation of the response to cis platinum treatment *in vitro*. *Int. J. Cancer*, **41**, 809.

BEDFORD, P., FICHTINGER-SCHEPMAN, A.M.J., SHELLARD, S.A. & 3 others (1988). Differential repair of platinum-DNA adducts in human bladder and testicular tumor continuous cell lines. *Cancer Res.*, **48**, 3019.

BEDFORD, P., WALKER, M.C., SHARMA, H.L. & 4 others (1987). Factors influencing the sensitivity of two human bladder carcinoma cell lines to cis-diamminedichloroplatinum(II). *Chem. Biol. Interact.*, **61**, 1.

DIJT, F.J., FICHTINGER-SCHEPMAN, A.M.J., BERENDS, F. & REEDIJK, J. (1988). Formation and repair of cisplatin-induced adducts to DNA in cultured normal and repair-deficient human fibroblasts. *Cancer Res.*, **48**, 6058.

D'INCALCI, M., SZMIGIERO, L., ERICKSON, L.C., HARTLEY, J.A. & KOHN, K.W. (1985). A filter incubation method for the determination of potentially crosslinkable sites in DNA in mammalian cells. *Anal. Biochem.*, **150**, 161.

DUCORE, J.M., ERICKSON, L.C., ZWELLING, L.A., LAURENT, G. & KOHN, K.W. (1982). Comparative studies of DNA cross-linking and cytotoxicity in Burkitt's lymphoma cell lines treated with cis-diamminedichloroplatinum (II) and L-phenylalanine mustard. *Cancer Res.*, **42**, 897.

EASTMAN, A. (1982). Comparison of the interaction of *cis*- and *trans*-diamminedichloroplatinum (II) with DNA by a simple filter binding assay. *Biochem. Biophys. Res. Commun.*, **105**, 869.

EASTMAN, A. (1985). Interstrand cross-links and sequence specificity in the reaction of cis-dichloro(ethylenediamine)platinum(II) with DNA. *Biochemistry*, **24**, 5027.

EASTMAN, A. (1987). Cross-linking of glutathione to DNA by cancer chemotherapeutic platinum coordination complexes. *Chem. Biol. Interact.*, **61**, 241.

EINHORN, L.H. & DONOHUE, J. (1977). Cis-diamminedichloroplatinum, vinblastine, and bleomycin combination chemotherapy in disseminated testicular cancer. *Ann. Int. Med.*, **87**, 293.

FICHTINGER-SCHEPMAN, A.M.J., VAN DER VEER, J.L., DEN HARTOG, J.H.J., LOHMAN, P.H.M. & REEDIJK, J. (1985*a*). Adducts of the antitumor drug cis-diamminedichloroplatinum(II) with DNA: formation, identification, and quantitation. *Biochemistry*, **24**, 707.

FICHTINGER-SCHEPMAN, A.M.J., BAAN, R.A., LUITEN-SCHUITE, A., VAN DIJK, M. & LOHMAN, P.H.M. (1985*b*). Immunochemical quantitation of adducts induced in DNA by cis-diamminedichloroplatinum(II) and analysis of adduct-related DNA-unwinding. *Chem. Biol. Interact.*, **55**, 275.

FICHTINGER-SCHEPMAN, A.M.J., VAN OOSTEROM, A.T., LOHMAN, P.H.M. & BERENDS, F. (1987). cis-diamminedichloropltinum(II)-induced DNA adducts in peripheral leukocytes from seven cancer patients: quantitative immunochemical detection of the adduct induction and removal after a single dose of cis-diamminedichloroplatinum(II). *Cancer Res.*, **47**, 3000.

GRUPPO INTERREGIONALE COOPERATIVO ONCOLOGICO GINECOLOGIA (1987). Randomised comparison of cisplatin with cyclophosphamide/cisplatin and with cyclophosphamide/doxorubicin/cisplatin in advanced ovarian cancer. *Lancet*, **ii**, 353.

KOHN, K.W., EWIG, R.A.G., ERICKSON, L.C. & ZWELLING, L.A. (1981). Measurement of strand breaks and cross-links by alkaline elution. In *DNA repair. A Laboratory Manual of Research Procedures*, Vol. **1**, Part B, Friedberg, E.C. & Hanawalt, P.C. (eds), p. 379. Marcel Dekker: New York.

LEE, F.Y.F., VESSEY, A., ROFSTAD, E., SIEMANN, D.W. & SUTHERLAND, R.M. (1989). Heterogeneity of glutathione content in human ovarian cancer. *Cancer Res.*, **49**, 5244.

LOEHRER, P.J. & EINHORN, L.H. (1984). Cisplatin. *Ann. Intern. Med.*, **100**, 704.

MICETICH, K., ZWELLING, L.A. & KOHN, K.W. (1983). Quenching of DNA: platinum(II) monoadducts as a possible mechanism of resistance to cis-diamminedichloroplatinum(II) in L1210 cells. *Cancer Res.*, **43**, 3609.

NEIJT, J.P., TEN BOKKEL HUININK, W.W., VAN DER BURG, M.E.L. & 8 others (1984). Randomised trial comparing two combination chemotherapy regimens (hexa-CAF *vs* CHAP-5) in advanced ovarian carcinoma. *Lancet*, **ii**, 594.

PERA, M.F. Jr, RAWLINGS, C.J. & ROBERTS, J.J. (1981). The role of DNA repair in the recovery of human cells from cisplatin toxicity. *Chem. Biol. Interact.*, **37**, 245.

PLOOY, A.C.M., VAN DIJK, M. & LOHMAN, P.H.M. (1984). Induction and repair of DNA cross-links in Chinese hamster ovary cells treated with various platinum coordination compounds in relation to platinum binding to DNA, cytotoxicity, mutagenicity, and antitumor activity. *Cancer Res.*, **44**, 2043.

POIRIER, M.C., LIPPARD, S.J., ZWELLING, L.A. & 6 others (1982). Antibodies elicited against cis-diamminedichloroplatinum(II)-modified DA are specific for cis-diamminedichloroplatinum(II)-DNA adducts formed *in vivo* and *in vitro*. *Proc. Natl Acad. Sci. USA*, **79**, 6443.

ROBERTS, J.J. & FRIEDLOS, F. (1981). Quantitative aspects of the formation and loss of DNA interstrand crosslinks in Chinese hamster cells following treatment with cis-diamminedichloroplatinum(II) (cisplatin). I. Proportion of DNA-platinum reactions involved in DNA crosslinking. *Biochim. Biophys. Acta*, **655**, 146.

SANCAR, A. & SANCAR, G.B. (1988). DNA repair enzymes. *Annu. Rev. Biochem.*, **57**, 29.

SUNDQUIST, W.I., LIPPARD, S.J. & STOLLAR, B.D. (1987). Monoclonal antibodies to DNA modified with cis- or trans- diamminedichloroplatinum(II). *Proc. Natl Acad. Sci. USA*, **84**, 8225.

VERMORKEN, J.B., VAN DER VIJGH, W.J.F., KLEIN, I. & 3 others (1984). Pharmacokinetics of free and total platinum species after short-term infusion of cisplatin. *Cancer Treat. Rep.*, **68**, 505.

WAUD, W.R. (1987). Differential uptake of cis-diamminedichloroplatinum(II) by sensitive and resistant murine L1210 leukemia cells. *Cancer Res.*, **47**, 6549.

WILTSHAW, E., SUBRAMARIAN, S., ALEXOPOULOS, C. & BARKER, G.H. (1979). Cancer of the ovary: a summary of experience with cis-dichlorodiammineplatinum(II) at The Royal Marsden Hospital. *Cancer Treat. Rep.*, **63**, 1545.

WOLF, C.R., HAYWARD, I.P., LAWRIE, S.S. & 6 others (1987). Cellular heterogeneity and drug resistance in two ovarian adenocarcinoma cell lines derived from a single patient. *Int. J. Cancer*, **39**, 695.

YOUNG, R., KNAPP, R.C., FUKS, Z. & DISAIA, P.J. (1985). Cancer of the ovary. In *Cancer: Principles and Practice of Oncology*, 2nd ed., De Vita, V.T., Hellman, S & Rosenberg, S.A. (eds), p. 1083, J.B. Lippincott: Philadelphia.

ZWELLING, L.A., ANDERSON, T. & KOHN, K.W. (1979*a*). DNA-protein and DNA interstrand cross-linking by cis- and trans-platinum(II) diamminedichloride in L1210 mouse leukemia cells and relation to cytotoxicity. *Cancer Res.*, **39**, 365.

ZWELLING, L.A., FILIPSKI, J. & KOHN, K.W. (1979*b*). Effect of thiourea on survival and DNA cross-link formation in cells treated with platinum(II) complexes, L-phenylalanine mustard, and bis(2-chloroethyl)methylamine. *Cancer Res.*, **39**, 4989.